# Tibio-pedal arterial pressure assessment during endovascular intervention to improve quality-of-life in patients with intermittent claudication

**DOI:** 10.3389/fcvm.2022.1038353

**Published:** 2022-11-29

**Authors:** Tak W. Kwan, Samuel Lee, Patricia Lin, Michael Liou, Henry Siu, Apurva Patel, Zoltan Ruzsa

**Affiliations:** ^1^Department of Cardiology, Lenox Health Greenwich Village, Northwell Health, New York, NY, United States; ^2^Department of Office-Based-Laboratory, Chinatown Cardiology, P.C., New York, NY, United States; ^3^Department of Medicine, Lincoln Memorial University–DeBusk College of Osteopathic Medicine, Harrogate, TN, United States; ^4^Department of Medicine, Lake Erie College of Osteopathic Medicine, Elmira, NY, United States; ^5^Department of Cardiology, Semmelweis University, Budapest, Hungary

**Keywords:** hemodynamics, tibio-pedal access, quality-of-life, pedal pressure, claudication, peripheral arterial disease

## Abstract

**Objective:**

The aim of this study is to compare the quality-of-life (QOL) outcomes and the tibio-pedal arterial pressure post-endovascular intervention.

**Background:**

Physiological assessment of peripheral arterial lesions is infrequently performed during endovascular interventions.

**Materials and methods:**

We retrospectively reviewed all 343 patients with intermittent claudication who underwent an endovascular intervention *via* tibio-pedal artery access from October 2018 to May 2021. The baseline and post-intervention tibio-pedal arterial pressures from the pedal sheaths were measured. QOL was assessed using a pre-validated Walking Impairment Questionnaire (WIQ) score before and at 30-day after intervention. We compared the baseline tibio-pedal arterial pressure, post-intervention tibio-pedal arterial pressure, delta pressure (post-intervention minus baseline), baseline WIQ scores, 30-day WIQ scores, and delta score (30-day minus baseline).

**Results:**

All 343 patients had successful tibio-pedal accesses. The average tibio-pedal arterial pressure at baseline was 87.0 ± 1.8 mmHg vs. 135.5 ± 1.7 mmHg post-intervention (*p* < 0.001). Average baseline and 30-day WIQ scores were summation (99.8 ± 3.3 vs. 115.0 ± 3.1, *p* < 0.001), walking distance (35.7 ± 1.3 vs. 42.5 ± 1.3, *p* < 0.001), walking speed (21.1 ± 0.9 vs. 23.6 ± 0.8, *p* = 0.036), stair climbing (4.7 ± 1.4 vs. 24.2 ± 1.4, *p* = 0.019), and symptoms (18.8 ± 0.2 vs. 20.1 ± 0.2, *p* < 0.001), respectively. When comparing the increased post-intervention tibio-pedal arterial pressure <60 mmHg vs. ≥60 mmHg, the average delta WIQ scores were all significantly improved with summation (10.0 ± 3.9 to 25.8 ± 5.5, *p* = 0.01), walking distance (4.1 ± 1.7 to 9.8 ± 2.5, *p* = 0.02), walking speed (1.5 ± 1.1 to 4.3 ± 1.5, *p* = 0.02), stair climbing (2.3 ± 1.8 to 9.4 ± 2.5, *p* = 0.02), and symptoms (1.0 ± 0.3 to 1.8 ± 0.4, *p* = 0.04), respectively.

**Conclusion:**

Increasing the post-intervention tibio-pedal arterial pressure by 60 mmHg can enhance QOL as suggested by improvement of WIQ scores.

## Introduction

Lower extremityperipheral arterial disease (PAD) is a chronic arterial occlusive disease caused by atherosclerosis, which results in diminished blood flow with a variety of symptoms such as reduced functional capacity, intermittent claudication, and critical limb ischemia. Previous studies have reported a prevalence of PAD related symptoms, mainly intermittent claudication, which ranges from 5.3 to 18.9% among the elderly population (1–4). Endovascular interventions of intermittent claudication are currently being used with an increased frequency because of the increased awareness and access of care and improvement of techniques and equipment (5–8). It is reasonable to believe that the objective of endovascular interventions is to restore blood flow from arterial blockage and ischemia, which would directly relate to adequate perfusion to the lower extremity muscles. Although it would be valuable information to understand the hemodynamics of lower extremity arteries before and after interventions, it has not been thoroughly investigated. There are only a few studies that address the measurement of pedal blood flow by using either invasive or non-invasive means in patients with intermittent claudication (9–16). Previous reports have demonstrated that tibio-pedal access (TPA) during endovascular interventions is safe and feasible (17–28). A recent randomized study of TPA showed advantages when compared to femoral or transradial accesses (24). Obtaining tibio-pedal arterial pressures from TPA is relatively simple and perfusion to the foot with increase of tibio-pedal arterial pressure can be easily assessed. Considering the infrequent use of quality-of- life (QOL) tools to assess outcomes in clinical practice, we believe that the measurement of tibio-pedal arterial pressures before and at the conclusion of interventions may be a promising approach to assess the outcome. Thus, in this report, we aim to investigate the QOL by evaluating the improvement of tibio-pedal arterial pressures for patients with intermittent claudication after endovascular interventions. The results of this study will be significant for understanding the physiology of peripheral circulation.

## Materials and methods

From October 2018 to May 2021, data was prospectively entered into a dedicated database to conduct a review of patients who were referred for the evaluation and treatment of intermittent claudication. All patients underwent peripheral interventions *via* TPA in this study. The protocol of the tibio-pedal retrograde approach for revascularization can be found in previous studies (18, 21). In brief, the TPA was chosen as the initial approach and performed by experienced operators. Under ultrasound guidance by an experienced technician, the flow of the dorsalis pedis artery, posterior tibial artery, or peroneal artery was demonstrated by Doppler in the short and long axis views. Either the dorsalis pedis artery, posterior tibial artery, or peroneal artery was accessed using a 21/19 tapered gauge echogenic tip needle with an anterior wall puncture technique followed by 4F Pinnacle Precision^®^ (Terumo Co.). Systemic heparin was given to maintain activated clotting time >300 s. An intra-arterial antispasmodic cocktail with combination of 100 μg of nitroglycerin, and 2.5 mg of verapamil were injected intra-arterially. If a significant lesion was identified, either atherectomy, balloon angioplasty, and/or stents were performed *via* the same or upsize Slender Glide sheath^®^ (Terumo Co.) from the retrograde approach at the discretion of the operator.

Baseline tibio-pedal arterial pressures from the tibio-pedal sheath were measured by a hemodynamic monitor after intra-arterial cocktail. The post-interventional tibio-pedal arterial pressure was measured after administration of nitroglycerin and before the sheath removal. The values were obtained in real-time and chosen as the most stable value.

We used the disease-specific, validated Walking Impairment Questionnaire (WIQ) that consists of four subcategories: walking distance, walking speed, stair climbing, and symptoms (29–32). All subcategories are scored from 0 (worst/inability) to 4 (best/without limitations). Walking distance score assessed the degree of difficulty in walking a specific distance, ranging from walking 50 feet to 1,500 feet or five blocks. In the walking speed score, the patients are asked to assess the degree of difficulty of walking a block in a specific speed (walking slowly to jogging). The stair climbing score reports the difficulty in climbing 1, 2, and 3 flights of stairs. The symptoms score describes the amount of difficulty with walking due to pain in lower extremities (one to three). The score is multiplied by a pre-specified weight for each question. WIQ was completed by patients and a trained nurse. It was assessed at two time points: pre-intervention (questionnaire was completed by patients when they were seen in the clinic) and at 30-day post-intervention (questionnaire completed by patients either in the clinic or verbally *via* the telephone).

We compared the delta tibio-pedal arterial pressures (post-intervention tibio-pedal arterial pressure minus pre-intervention tibio-pedal arterial pressure) and the delta WIQ score, summation, and subcategories (30-day post-intervention WIQ score minus pre-intervention WIQ score) in this cohort of patients. When using trial and error method with increment of 10 mmHg of the increased post-intervention tibio-pedal arterial pressure to compare the delta WIQ scores, we found that 60 mmHg was the best cutoff point for statistical significance.

Continuous variables were compared using two-sample unpaired *t*-tests. A *p*-value of 0.05 was considered the threshold of statistical significance. The protocol was approved by the local institutional review board and informed consents were obtained in all patients for procedure only but not for enrollment of this study.

## Results

**FIGURE 1:**
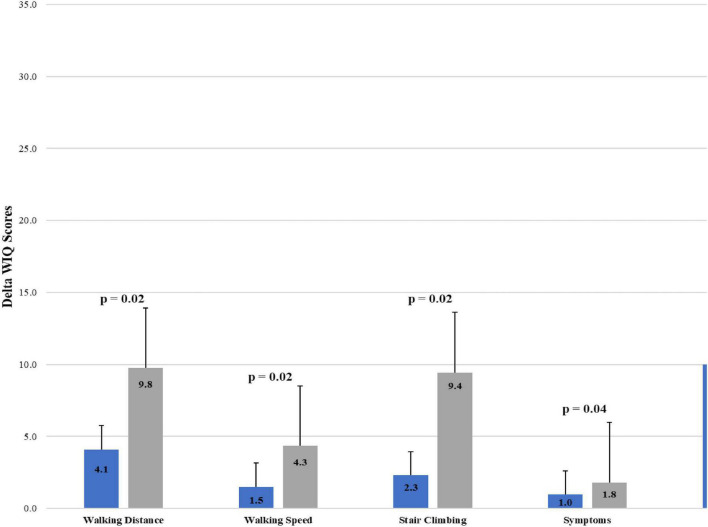
Comparing the delta walking impairment questionnaire (WIQ) scores (summation, walking distance, walking speed, stair climbing, symptoms) to increase of tibio-pedal arterial pressures > or ≤60 mmHg after intervention. Delta Walking Impairment Questionnaire (WIQ) Scores = 30-day post-intervention score minus pre-intervention score.

A total of 343 consecutive patients underwent retrograde endovascular intervention *via* TPA for intermittent claudication was identified from the study. Patient demographics are summarized in Table 1. Majority of the patients are male (60%), and hypertension was found in 87% of patients. Procedural characteristics are shown in Table 2. Successful TPA were obtained in 100% of patients. Dorsalis pedis/Anterior tibial artery was the most common access site (72%). Table 3 shows the average tibio-pedal arterial pressures and average WIQ summation and subcategory scores before and after interventions. Average tibio-pedal arterial pressure at baseline was 87.0 ± 1.8 mmHg vs. 135.5 ± 1.7 mmHg post-intervention (*p* < 0.001). Average baseline and 30-day WIQ scores were summation (99.8 ± 3.3 vs. 115.0 ± 3.1, *p* < 0.001), walking distance (35.7 ± 1.3 vs. 42.5 ± 1.3, *p* < 0.001), walking speed (21.1 ± 0.9 vs. 23.6 ± 0.8, *p* = 0.036), stair climbing (4.7 ± 1.4 vs. 24.2 ± 1.4, *p* = 0.019), and symptoms (18.8 ± 0.2 vs. 20.1 ± 0.2, *p* < 0.001), respectively. When comparing the increased post-intervention tibio-pedal arterial pressure <60 mmHg vs. ≥60 mmHg, the average delta WIQ scores were all significantly improved with summation (10.0 ± 3.9 to 25.8 ± 5.5, *p* = 0.01), walking distance (4.1 ± 1.7 to 9.8 ± 2.5, *p* = 0.02), walking speed (1.5 ± 1.1 to 4.3 ± 1.5, *p* = 0.02), stair climbing (2.3 ± 1.8 to 9.4 ± 2.5, *p* = 0.02), and symptoms (1.0 ± 0.3 to 1.8 ± 0.4, *p* = 0.04), respectively (Figure 1).

**TABLE 1:** Baseline patient characteristics.

	*n* = 343
Age (yr.)	76 ± 10.5
Male (%)	207 (60)
Hypertension (%)	299 (87)
Diabetes (%)	177 (34)
Hyperlipidemia (%)	264 (77)
Smoking (%)	178 (52)
Atrial fibrillation (%)	60 (17)
Coronary artery disease (%)	255 (75)
Myocardial infarction (%)	11 (3)
Cerebral vascular accident (%)	67 (20)
Creatinine	1.3 ± 1.1
Rutherford class 2	38 (11)
Rutherford class 3	305 (89)

**TABLE 2:** Procedural characteristics.

Fluoroscopy time (sec.)	1038.8 ± 829.7
Contrast volume (cc.)	36.7 ± 8.8
Radiation dosage (mGy)	108.4 ± 149.7
Successful pedal artery puncture (%)	100
DP/AT artery (%)	72
PT artery (%)	14
Peroneal artery (%)	14
TASC (Fem-Pop) (%)	
A	8
B	28
C	42
D	22
TASC (Tibial) (%)	
A	10
B	21
C	59
D	10

**TABLE 3:** Comparing the average tibio-pedal arterial pressures values, and average walking impairment questionnaire scores at baseline and post-intervention.

	Baseline	Post-intervention	*P*-value
Tibio-pedal arterial pressure (mmHg)	87.0 ± 1.8	135.5 ± 1.7	<0.001
**Walk impairment questionnaire**			
Summation score	99.8 ± 3.3	115.0 ± 3.1	<0.001
Walking distance score	35.7 ± 1.3	42.5 ± 1.3	<0.001
Walking speed score	21.1 ± 0.9	23.6 ± 0.8	0.036
Stair climbing score	4.7 ± 1.4	24.2 ± 1.4	0.019
Symptoms score	18.8 ± 0.2	20.1 ± 0.2	<0.001

## Discussion

Improving patients’ symptoms and daily function is the main objective in endovascular revascularization treatment for patients with intermittent claudication. Theoretically, after successful endovascular intervention, the perfusion to the lower extremity muscle is improved and should be evidenced by the improvement of direct tibio-pedal arterial pressure measurement. The current study demonstrated that (1) endovascular revascularization treatment resulted in significant improvements in both tibio-pedal arterial pressures and subjective QOL outcomes assessed by WIQ. (2) Patients with a post-intervention increased of tibio-pedal arterial pressure ≥60 mmHg showed a greater improvement in walking distance, walking speed, climbing stairs, or symptoms.

From several landmark coronary intervention studies (33–35), angiography can only provide the anatomical information but not the functional hemodynamics data of the lesions before and after revascularization. Several small studies of invasive hemodynamics assessments in peripheral arteries have been done previously (9–11, 15, 16, 36). In general, the site of arterial access from most of the studies is typically from the femoral artery or occasionally from the radial artery. Not only did they all have a small number of patients, but they were also using a pressure wire or catheters to assess the arterial pressure gradients. Recently, we have developed a novel and simple approach that allows tibio-pedal arterial pressure assessment from TPA for patients during retrograde endovascular interventions (9, 10). This approach might give us more accurate information for hemodynamics assessment and perfusion to the lower extremities than the anatomical information from peripheral angiography.

Recent trials have assessed intermittent claudication-related symptoms and functional impairment using the WIQ, which mainly focuses on the patients’ walking distance and functioning ability (29–31). The WIQ demonstrated validity and reliability in patients with PAD. In the study of Myers et al. it was reported that QOL limitations are most closely related to claudication and reflect the validated WIQ (30). As WIQ score increases and with improved QOL, there is also a positive trend of survival and clinical cardiovascular benefit (37, 38). From a large study with 1,048 patients, Jain et al. (37) suggested that only the stair-climbing score predicted all-cause and cardiovascular mortality with PAD but not the walking distance or speed Scores. However, Nead et al. (32) studied 1,417 precipitants with or without PAD, they found that walking distance, speed, and stair-climbing scores all independently predicted future all-cause and cardiovascular mortality, Further studies will be needed to clarify the findings.

In this study, after endovascular interventions, an increase in tibio-pedal arterial pressure of at least 60 mmHg signify an improvement of WIQ scores at 30-days. This finding has significant implications for endovascular interventions in patients with peripheral arterial disease. The improvement of tibio-pedal arterial pressure not only correlates with limb salvage in patients with critical limb ischemia (10) but we can also expect an improvement of QOL in patients with intermittent claudication. The definition for successful endovascular intervention should no longer be a reduction of anatomic diameter of the lesions but it should also include an improvement of hemodynamic pressure gradient. A physiological assessment should be part of the investigation in endovascular intervention of peripheral arterial disease.

## Limitations

We revealed several limitations in this study. (1) The study is not a randomized study and subjects to selection bias. Furthermore, not all information including ultrasound data is available for study. (2) The tibio-pedal arterial pressure was obtained invasively from tibio-pedal access only. (3) The interval between revascularization and the post-revascularization assessment of treatment was relatively short. To understand restenosis or disease progression affecting WIQ scores will require further study. (4) We only showed the improvement of QOL with improvement of delta tibio-pedal arterial pressure by at least 60 mmHg. However, correlation analysis did not show significance.

## Conclusion

The benefits of QOL in patients with intermittent claudication may be closely related to improvements in the tibio-pedal arterial pressures after revascularization. Our data suggests that patients reported improvement in PAD symptoms following lower extremity revascularization is indeed associated with post-revascularization improvements of tibio-pedal arterial pressures ≥60 mmHg. Among patients with intermittent claudication, measuring pedal pressures can provide a simple, feasible, and reliable tool for endovascular interventions. Future long-term and larger study should validate the increased of post-intervention tibio-pedal arterial pressure of 60 mmHg and to address restenosis in treating patients with intermittent claudication.

## Data availability statement

The raw data supporting the conclusions of this article will be made available by the authors, without undue reservation.

## Ethics statement

The studies involving human participants were reviewed and approved by Western Institutional Review Board. Written informed consent for participation was not required for this study in accordance with the national legislation and the institutional requirements.

## Author contributions

TK: conceptualization, methodology, writing, and supervision. SL and PL: data collection, statistical analysis, and editing. ML: data collection and supervision. HS and AP: writing and data collection. ZR: conceptualization and methodology. All authors contributed to the article and approved the submitted version.

## Abbreviations

QOL: quality-of-life
WIQ: walking impairment questionnaire
TPA: tibio-pedal access
PAD: peripheral arterial disease.
